# Delirium Increases Risk of Mild Cognitive Impairment (MCI) and Dementia in the Mayo Clinic Study of Aging

**DOI:** 10.1093/geroni/igaf122.4292

**Published:** 2025-12-31

**Authors:** Allyson Palmer, Aidan Mullan, Heling Jia, Sunghwan Sohn, Fernanda Bellolio

**Affiliations:** Mayo Clinic, Rochester, Minnesota, United States; Mayo Clinic, Rochester, Minnesota, United States; Mayo Clinic, Rochester, Minnesota, United States; Mayo Clinic, Rochester, Minnesota, United States; Mayo Clinic, Rochester, Minnesota, United States

## Abstract

Delirium is a state of acute confusion characterized by deficits in attention that occurs commonly in hospitalized older adults and is associated with adverse outcomes. Delirium may have significant implications for cognitive health, however the relationship of delirium to mild cognitive impairment (MCI) and dementia is not fully understood. To evaluate the impact of delirium on cognitive health trajectories, we leveraged the Mayo Clinic Study of Aging (MCSA), a population-based cohort study of cognitive health and aging in residents of Olmsted County, Minnesota, who are between the ages of 30 and 89 and without a previous diagnosis of dementia at enrollment. Emergency Department and hospital encounters were identified for all patients enrolled in the MCSA, and clinical notes following each encounter up to 30 days were analyzed using a previously validated natural language processing (NLP) algorithm for delirium detection. The association between delirium within 30 days of hospitalization and the development of MCI or dementia was assessed using Cox proportional hazards regression, adjusted for patient age, sex, and level of education. Initial MCSA assessment indicated normal cognition for all individuals in this cohort. We found that delirium was significantly associated with development of MCI and dementia, with 42 individuals experiencing delirium within 30 days of hospitalization; hazard ratio of 10.07 (95% CI 4.14, 24.49) for developing MCI and 19.10 (95% CI 8.40, 43.47) for dementia compared to those without delirium. Our results suggest that hospitalization with an episode of delirium can significantly impact cognitive trajectories in older adults.

